# Quinoa genotypes under deficit irrigation: integrating phenotyping and remote sensing for water use efficiency in arid Peru

**DOI:** 10.3389/fpls.2026.1810225

**Published:** 2026-08-11

**Authors:** Raquel Shany Betancur-Choquehuayta, Esther Emiliana Molina-Gonzales, Dony Javier Flores-Subeleta, Raymundo O. Gutiérrez-Rosales, Alberto Anculle-Arenas, Natty Wilma Llasaca-Calizaya, José Luis Bustamante-Muñoz, Mayela Elizabeth Mayta-Anco

**Affiliations:** 1 Facultad de Agronomía, Universidad Nacional de San Agustín de Arequipa, Arequipa, Peru; 2 Facultad de Ciencias de Ingeniería, Universidad de Huánuco, Huánuco, Peru; 3 Facultad de Ciencias Histórico Sociales, Universidad Nacional de San Agustín de Arequipa, Arequipa, Peru

**Keywords:** deficit irrigation, quinoa, remote sensing, vegetation index, water use efficiency

## Abstract

Within the context of climate change, quinoa (*Chenopodium quinoa* Willd.) is a climate-resilient crop with high nutritional value. The effects of deficit irrigation on quinoa growth and physiological performance under arid conditions remain insufficiently understood. This study evaluated ten quinoa genotypes (two commercial varieties and eight accessions) under two irrigation regimes to identify traits and spectral indices associated with water-stress tolerance. We combined manual phenotyping of agromorphological and physiological traits with multispectral and spectroradiometer measurements to calculate 35 vegetation indices across 13 and 5 dates, respectively. Deficit irrigation reduced plant height (18%), specific leaf area (8%), yield (43%), harvest index (26%), relative water content (7%), and dry matter accumulation (36%), while relative chlorophyll content (SPAD, Soil Plant Analysis Development) and stomatal density increased by 16% and 13%, respectively; accession ACC_23 exhibited the highest water-use efficiency (5.9 g kg^-1^). A univariate analysis of 35 vegetation indices across 13 dates showed that: Health Index(HIV), Normalized Green-Red Difference Index (NGRD), Red-Green Ratio (RG) and Plant Senescence Reflectance Index (PSRI), were the most sensitive, detecting significant differences between irrigation treatments in up to 32 of the 130 possible genotype-by-date comparisons. Integrating remote sensing into crop phenotyping represented a significant methodological improvement by enhancing phenotyping efficiency, improving detection of deficit irrigation effects, and facilitating identification of tolerant quinoa genotypes for arid production systems.

## 1 Introduction

Water stress in plants, intensified by climate change, represents one of the main constraints to agriculture, significantly affecting crop productivity and threatening global food security (Prajapati et al., 2024; Yuan et al., 2024). This challenge is particularly severe in arid and semi-arid regions, where irrigation water availability is becoming increasingly limited and farmers are forced to optimize irrigation management and select crops with greater water-use efficiency (Dinar et al., 2019; Asprilla-Echeverria, 2024; Rao et al., 2024).

Under deficit irrigation conditions, plants undergo several physiological and morphological alterations that affect growth and productivity. One of the earliest responses is the reduction in water uptake and leaf water potential, leading to decreased tissue turgor and cellular dehydration (Jaramillo Roman et al., 2020; Aktas et al., 2025), Water deficit also restricts cell expansion and division, reducing leaf area development and stem growth (Nadali et al., 2021; Mirsafi et al., 2024; Aktas et al., 2025) In addition, stomatal closure decreases transpiration and CO_2_ assimilation, negatively affecting photosynthetic activity, electron transport, and chlorophyll-related processes (Manaa et al., 2021; Saddiq et al., 2021).

These physiological responses ultimately reduce biomass accumulation and crop yield under prolonged deficit irrigation conditions. In this context, improving water-use efficiency and identifying crops tolerant to deficit irrigation have become priorities for agricultural research programs (El-Harty et al., 2023; Mirsafi et al., 2024) Quinoa (*Chenopodium quinoa* Willd an Andean pseudocereal, has emerged as a strategic crop due to its remarkable adaptability to adverse environmental conditions, including salinity, frost, and limited water availability (De La Cruz-Arango, 2023) as well as its outstanding nutritional profile (Chaudhary et al., 2023; Manzanilla-Valdez et al., 2024). In addition, quinoa exhibits several physiological and biochemical adaptation mechanisms, such as osmotic adjustment, antioxidant accumulation, and stress-related protein synthesis, which contribute to tolerance under deficit irrigation conditions (Hariadi et al., 2011; Ruiz-Carrasco et al., 2011). These characteristics make quinoa an attractive crop for agricultural systems increasingly affected by climate change and water limitations (Bazile et al., 2016; Campbell et al., 2016).

Traditional phenotyping approaches in plant breeding rely on direct measurements of agromorphological and physiological traits, which are often labor-intensive, destructive, and limited in spatial and temporal scale. In this context, remote sensing technologies provide non-invasive, accurate, and scalable alternatives for crop monitoring (Zhang and Kovacs, 2012; Araus and Cairns, 2014; Lupa-Condo et al., 2024). Remote sensors capture spectral information associated with plant physiological status, enabling early detection and evaluation of crop responses to deficit irrigation through vegetation indices (VIs) derived from algebraic combinations of spectral bands at different wavelengths. Vegetation indices such as NDVI (Normalized Difference Vegetation Index), EVI (Enhanced Vegetation Index), and PRI (Photochemical Reflectance Index) have demonstrated strong potential for monitoring crop physiological responses under water-deficit conditions (Su et al., 2023; Wang et al., 2023; Wu et al., 2023; Liu et al., 2024).

While remote sensing technologies have been used to monitor crop water status, their application for the selection and characterization of genetic diversity in quinoa responses to deficit irrigation, particularly under the specific agroecological conditions of the Peruvian coast, remains poorly explored (Geerts, 2008; Talebnejad and Sepaskhah, 2015; Lupa-Condo et al., 2024; Mirsafi et al., 2024). Furthermore, the integration of multispectral imagery with detailed agromorphological and physiological measurements to identify vegetation indices capable of early stress detection (Cocozza et al., 2025; Faqeerzada et al., 2025; Muhammad et al., 2025), and the selection of tolerant genotypes represents an important step for crop improvement programs (Saddiq et al., 2021; Mousa et al., 2025).

The primary objective of this study was to evaluate the effects of deficit irrigation on ten quinoa genotypes (two commercial varieties and eight genotypes) grown in the arid zone of Majes, Arequipa, Peru, through biometric and remote sensing measurements. The specific objectives were: (i) to identify genotypes with superior agromorphological and physiological characteristics due to the effect of irrigation treatment; (ii) determine tolerance indices for deficit irrigation; (iii) evaluate water use efficiency (WUE) among genotypes; (iv) to determine the vegetation indices capable of detecting deficit irrigation; (v) to characterize the spectral signatures of the quinoa genotypes under deficit irrigation and (vi) to determine the statistical interrelation of the variables evaluated.

## 2 Material and methods

### 2.1 Study area

**Figure 1:**
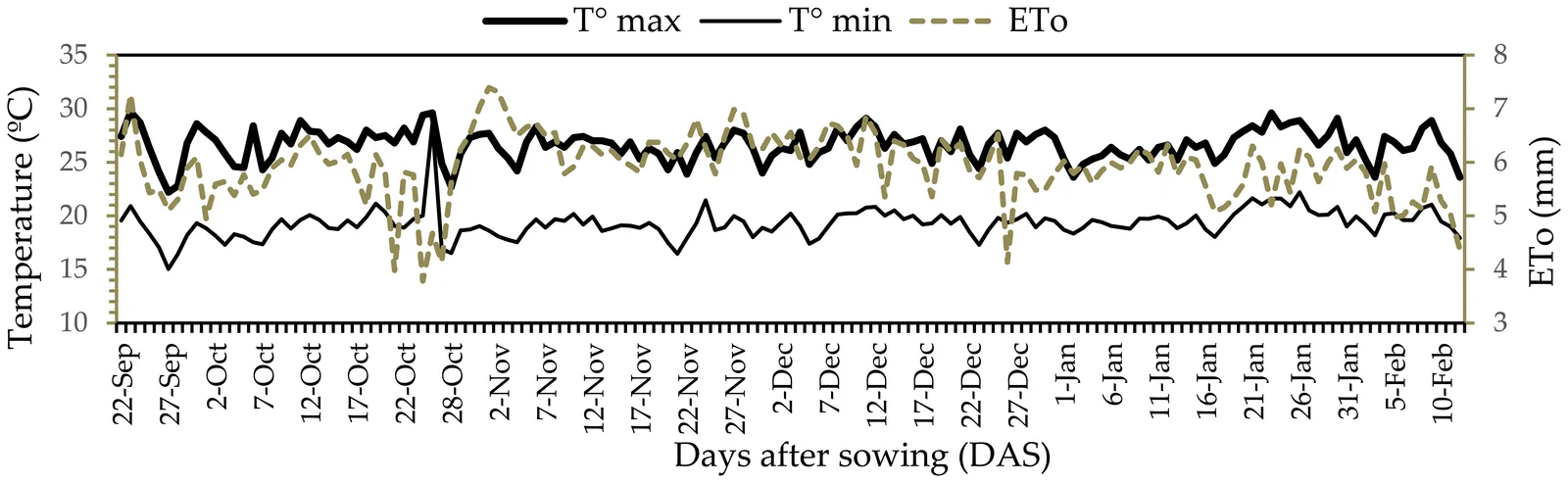
Daily maximum temperatures (T max, thick line), minimum temperatures (T min, thin line) and reference evapotranspiration (ETo, dashed line), during crop development (2023) until harvest (2024).

The study was carried out in the Majes Irrigation (16°19’38.820”S, 72°13’1.812” W, altitude of 1428 meters above sea level) of the experimental field of the CIEPA (Center for Agricultural Research, Teaching and Production), Majes, Arequipa, Peru. The soil of Majes Irrigation has a sandy loam texture and other soil properties are shown in **Table 1**. The daily climatic data (rainfall, wind speed, relative humidity, solar radiation, temperature and evapotranspiration), used in this study were obtained from a Davis Vantage Pro2 Plus meteorological station (Davis Instruments Corporation: Hayward, CA, USA), operated by AUTODEMA (Autonomous Authority of Majes), located 3.08 km from the experimental plot. During the crop season, minimum and maximum temperatures of 10.1 °C and 29.3 °C, respectively, were recorded, as shown in **Figure 1**. In addition, daily monitoring of the reference evapotranspiration (ET_o_) was carried out, which allowed the development of irrigation programs.

**Table 1:** Soil properties of the experimental field.

Texture (%)			pH	E.C. (mS m^-1^)	O.M. (%)	P (mg kg^-1^)	K (mg kg^-1^)
Sand	Clay	Silt					
76.80	10.50	12.70	8.00	55.88	1.10	31.20	487.69

E.C, Electrical Conductivity; O.M., Organic Matter; P: Phosphorus; K, Potassium.

### 2.2 Experimental design and treatments

The experimental field covered a total area of 1512 m^2^ and was divided into two plots of 684 m^2^ each, separated by a 4 m buffer zone. Each plot comprised 50 experimental units of 10.8 m^2^. The experiment was arranged in a randomized complete block design (RCBD) with five replicates to evaluate the effects of different irrigation regimes on ten quinoa genotypes (**Table 2**). The irrigation treatments were applied independently in each plot according to the parameters described in **Table 3**, up to 47 days after sowing, both treatments had normal irrigation; after that, irrigation was differentiated. Manual sowing was carried out on September 22, 2023, in each experimental unit they were planted in four rows with a spacing of 0.9 m between rows and 0.2 m between strokes (stroke = set of 10 plants), resulting in a density of 50 plants per linear meter.

**Table 2:** Genetic material utilized in this experiment.

Nro.	Genotype ID	GB UNSA Code ^1^
1	SALCEDO	Salcedo INIA
2	B_JULY	Blanca de Juli
3	ACC_50	UNSA-CH-1900050
4	ACC_49	UNSA-CH-1900049
5	ACC_44	UNSA-CH-1900044
6	ACC_43	UNSA-CH-1900043
7	ACC_32	UNSA-CH-1900032
8	ACC_23	UNSA-CH-1900023
9	ACC_20	UNSA-CH-1900020
10	ACC_06	UNSA-CH-1900006

^1^ GB UNSA CODE: Code provided by the Laboratorio del Banco de Germoplasma Project of the Universidad Nacional de San Agustín de Arequipa.

**Table 3:** Volume of water applied per plot.

Quinoa plot	Treatment	Water Requirement (WR)	Water volume (m^3^ ha^-1^)
1	Normal Irrigation (NI)	100%	6, 272.7
2	Deficit Irrigation (DI)	50%	3, 593.6

Irrigation was administered using a drip irrigation system controlled by Dream Spot software (Talgil Computing & Control Ltd., Kiryat Motzkin, Israel) (Dream Console, n.d.).This system allowed two daily irrigation cycles, which were adjusted according to the phenological stage of the crop (Allen et al., 1998) to optimize water use according to the requirements of each treatment. The crop water requirement was determined using the Penman-Monteith equation (Zotarelli et al., 2024):


$$ET_{c}=K_{c}*ET_{o}$$

Donde;

ET_c_ crop evapotranspiration [mm d^-1^].

K_c_ crop coefficient.

ET_o_ reference crop evapotranspiration [mm d^-1^].

The daily irrigation volume was determined from ET_c_ and the area of each treatment, according to Allen et al. (1998). The Normal Irrigation (NI) treatment received 100% of the calculated daily ET_c_, whereas the Deficit Irrigation (DI) treatment received 50% of the daily ET_c_ calculated for NI. This reduction was applied consistently throughout the entire crop cycle. Crop coefficient (K_c_) values remained constant for both treatments, and only the irrigation water volume was reduced in the DI treatment. Fertilization was then applied based on the following nutrient rates per hectare: N, P_2_O_5_, K_2_O, CaO, and MgO (314.3, 358.2, 117.0, 51.2, and 43.9 kg ha^-1^, respectively), according to the soil analysis results shown in **Table 1**.

### 2.3 Agromorphological data acquisition

Genotypes were evaluated from 40 days after sowing (DAS), with several key measurements taken. Plant height (PH) was measured from the root neck to the apex of the panicle using a graduated ruler at 41, 55, 69, 85, and 95 DAS. Leaf area was determined from scanned JPG (joint photographic groups) format images using ImageJ software (Rasband, 2012). Subsequently, leaves were dried to obtain their dry mass, which was used to calculate Specific Leaf Area (SLA) as the ratio of leaf area to leaf dry mass (cm^2^ g^-1^). These measurements were performed at 60, 76, and 98 DAS.

At physiological maturity, which varied depending on the genotype between 106 and 143 DAS, data were taken from performance components such as panicle length (PL), which was measured from the base to the main apex of the panicle with a tape measure, following the descriptor measurement protocol of (Bioversity International, FAO, PROINPA, INIAF, and FIDA, 2013) and the panicle diameter (PD), which was also measured at its widest point of each panicle with a digital caliper at three different points.

After the final harvest (staggered between 106 and 143 DAS), the yield (Y), harvest index (HI), and thousand-grain weight (TGW) per experimental unit were calculated using a precision analytical balance (Radwag, Radom, Poland) of 0.0001 g. The grain diameter (GD) of 10 randomly obtained seeds was measured using a stereoscope (Euromex BV, Arnhem, Netherlands) and grain thickness (GT) was determined with the digital micrometer (Shahe, Ningbo, China).

### 2.4 Acquisition of physiological data

The physiological data of each genotype were evaluated at different times. The relative chlorophyll content was performed according to the procedure (Yongli Zhu et al., 2011), using a chlorophyll SPAD-502 meter (Konica Minolta Corp., Osaka, Japan), on leaves from the upper third of the plant during the nine dates: 40, 48, 54, 61, 68, 77, 82, 91 and 103 DAS. The relative water content (RWC) was measured according to the Barrs and Weatherley method (Barrs and Weatherley, 1962) on the dates of 60, 76 and 98 DAS, and was calculated using the formula:


$$RWC=\left(\frac{FW-DW}{TW-DW}\right)*100$$

Where

FW is initial leaf fresh weight [g],

TW is the leaf turgid weight [g] and

DW is the leaf dry weight [g].

Dry matter (DM) was calculated at 45, 60, 75 and 90 DAS, and at the time of harvest, extracting three complete plants per experimental unit, separating the aboveground biomass and roots, which were dried at 80 °C in an oven (HERAEUS, Hanau, Germany), for 48 hours and then weighed.

Stomatal density (SD) was assessed according to (Wilkinson, 1979). Three fully expanded leaves from the upper third of the plant were sampled per experimental unit. Impressions were obtained from the abaxial surface using ethyl acetate. Stomatal counts were performed in a single field of view (0.16 mm^2^) per leaf, located in the central region of the lamina between secondary veins, avoiding major veins and leaf margins. Counts were carried out using Image Focus Alpha software v.1.3.7.27179 (Image Focus Alpha, n.d.) with a trinocular microscope (AmScope, Irvine, USA) at 40× magnification. The average of the three leaves per experimental unit was used to estimate stomatal density. Measurements were conducted at 73 and 99 DAS.

### 2.5 Calculation of stress tolerance indices

The methodology to determine the seven indices of tolerance to deficit irrigation for the genotypes was based on the estimation of the equations in **Table 4** that allowed for calculation and identification of those genotypes that showed high stability and yield under conditions of deficit irrigation.

**Table 4:** Calculation of indices for evaluating tolerance to water stress.

Stress tolerance index	Outcome	Formula	Reference
Geometric Mean Productivity (GMP)	+ (T)/- (S)	$GMP=\sqrt{Yp*Ys}$	(Fernandez, 1992)
Harmonic Mean (HM)	+ (T)/- (S)	$HM=\frac{2*\left(Yp*Ys\right)}{\left(Yp+Ys\right)}$	(Chakherchaman et al., 2009)
Stress Tolerance Index (STI)	+ (T)/- (S)	$STI=\frac{\left(Yp*Ys\right)}{\bar{Y}p^{2}}$	(Fernandez, 1992)
Stress Tolerance Index (TOL)	- (T)/+ (S)	TOL=Yp−Ys	(Hossain et al., 1990)
Stress Intensity (SI)	- (T)/+ (S)	$SI=1-\left(\frac{\bar{Y}s}{\bar{Y}p}\right)$	(Fernandez, 1992)
Stress Susceptibility Index (SSI)	- (T)/+ (S)	$SSI=\frac{\left(1-\frac{Ys}{Yp}\right)}{SI}$	(Fernandez, 1992)
Stress and Non-Stress Production Index (SNPI)	+ (T)/- (S)	$SNPI=\sqrt{\frac{\left(Yp+Ys\right)}{\left(Yp\;-\;Ys\right)}}*\sqrt{Yp*Ys*Ys}$	(Mousavi et al., 2008)

T, Tolerant, outcome depending on the value (-) or (+) for each index; S, Susceptible, outcome depending on the value (-) or (+) for each index; *Yp*, mean yield of genotypes under non-stress conditions; *Ys*, mean yield of genotypes under stress conditions; $\bar{Y}p$, mean yield of all genotypes under non stress conditions; $\bar{Y}s$, mean yield of all genotypes under stress conditions.

### 2.6 Water use efficiency

The water use efficiency (WUE) was determined based on the dry matter measurements (DM) obtained at 45, 60, 75 and 90 DAS, measuring three entire plants per experimental unit according to (Sinclair et al., 1984) and the quantity of applied water, based on the following equation:

WUE= Dry matter [t ha^-1^]/amount of water applied [m^-3^].

### 2.7 Vegetation indices

**Figure 2:**
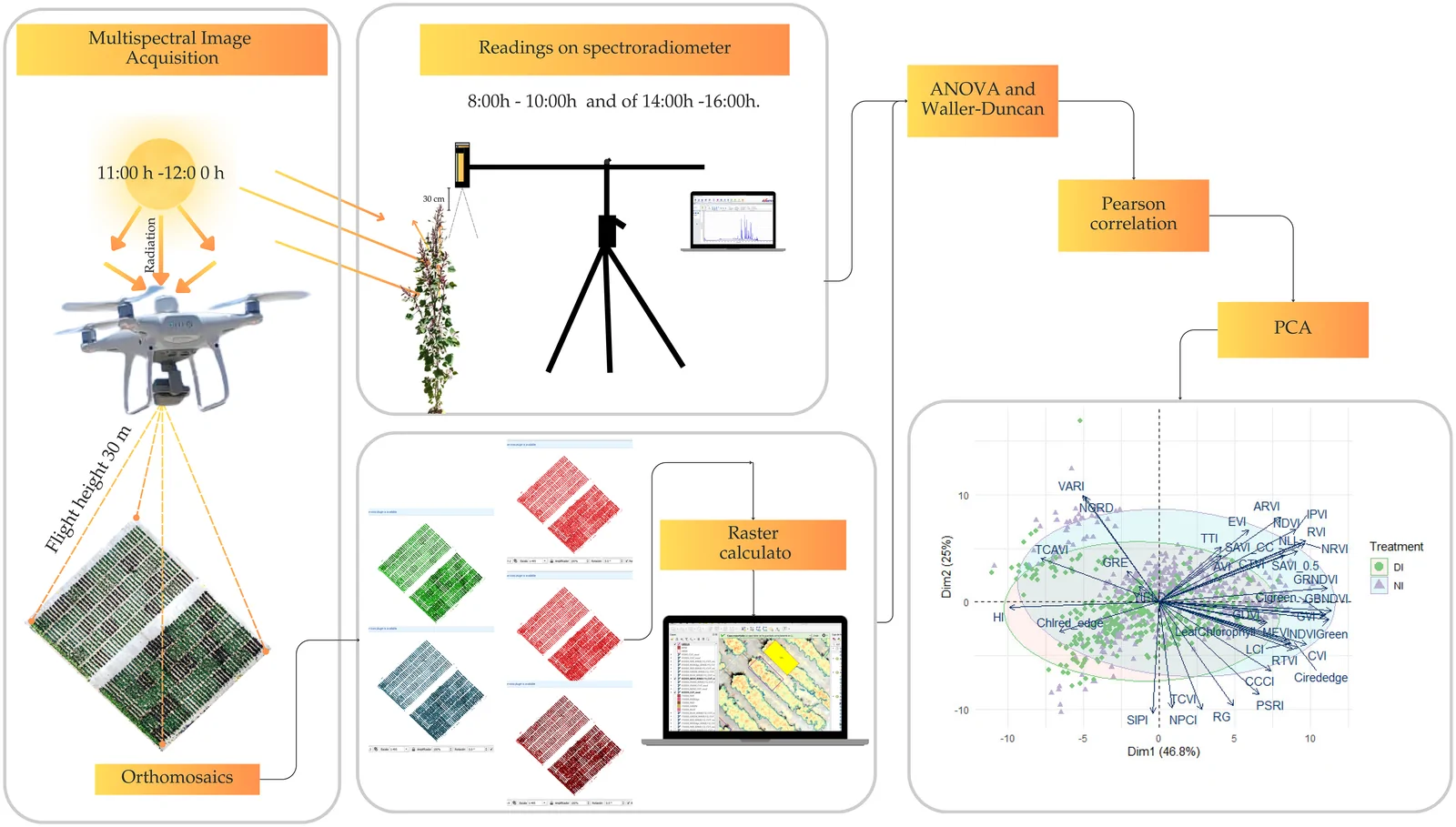
Flowchart of remote sensing data acquisition.

In this study, a Multispectral Phantom 4 camera (DJI, Shenzhen, China) was used, composed of five non-continuous spectral bands: blue (B) at 450 nm ± 16 nm, green (G) at 560 nm ± 16 nm, red (R) at 650 nm ± 16 nm, red edge (RE) at 730 nm ± 16 nm, and near infrared (NIR) at 840 nm ± 26 nm. The flight plan was carried out at a height of 30 m above the ground and executed approximately between 11:00 h and 12:00 h. Multispectral images were captured over a 2.0 s time interval with 75% frontal and lateral overlap at a spatial resolution of 1.6 cm pixel^-1^. **Figure 2** presents the methodology used in remote sensing.

An Agisoft Metashape v.2.0.3 photogrammetry software was used to generate orthomosaics from the multispectral images. Subsequently, according to the orthomosaics were aligned in the Quantum Geographic Information System (QGIS) v.3.28.12 using four control points previously established in the experimental field. Once the orthomosaics were aligned, the region of interest (ROI) was designated and a grid was generated on the central rows of each experimental unit for labeling. Soil and vegetation were segmented using an OSAVI threshold of 0.11 to isolate vegetation cover.

The vector data resulting from the labeling was converted to raster format, and a labeled mask was generated using the rasterization tool in QGIS. The average pixel values were recorded for each grid. Based on these values, 35 vegetation indices were calculated as shown in **Table 5**. In order to evaluate the discriminant sensitivity of these indices, a univariate analysis of the 35 VIs was performed using the student’s t-test. This procedure compared the pairwise means between treatments for each of the 13 evaluation times or dates, revealing the number of significant differences specific to each genotype. Finally, to prioritize the detection capacity of each spectral tool, the VIs were classified according to the frequency of statistical differences observed throughout the crop (Ihuoma and Madramootoo, 2019, Ihuoma and Madramootoo, 2020).

**Table 5:** Vegetation indexes (VIs).

Nro.	Abbrev.	Name	Formula	Primary sensitivity	Reference
1	NDVI	Normalized Difference Vegetation Index	$NDVI=\frac{\left(NIR-RED\right)}{\left(NIR+\;RED\right)}$	Biomass, greenness, and canopy structure	(Rouse et al., 1974)
2	TTI	Transformed Vegetation Index	$TTI={\left(\frac{\left(NIR-RED\right)}{NIR+RED}+0.5\right)}^{0.5}$	Biomass and vegetation cover	(Bannari et al., 1995)
3	RVI	Ratio Vegetation Index	$RVI=\frac{NIR}{RED}$	Biomass, Leaf Area Index (LAI), and chlorophyll	(Birth and McVey, 1968)
4	NRVI	Normalized Ratio Vegetation Index	$NRVI=\frac{RVI-1}{RVI+1}$	Biomass and greenness	(Lemenkova, 2020)
5	SAVI-CC	Soil-Adjusted Vegetation Index with Canopy Cover	$SAVI-CC=\left(\frac{NIR-RED}{NIR+RED+CC}\right)*\left(1+CC\right)$	Biomass (minimizes soil background interference)	(Huete et al., 1992)
6	SAVI-0.5	Soil-Adjusted Vegetation Index with L factor of 0.5	$SAVI_{0.5}=\left(\frac{NIR-RED}{NIR+RED+0.5}\right)*\left(1+0.5\right)$	Biomass and soil background reduction	(Shibayama et al., 1999)
7	AVI	Advanced Vegetation Index	AVI=NIR−RED	Biomass and vegetation density	(Liang et al., 2014)
8	IPVI	Infrared Percentage Vegetation Index	$IPVI=\frac{NIR}{\left(NIR+RED\right)}$	Biomass and vegetation vigor	(Crippen, 1990)
9	CTVI	Corrected Transformed Vegetation Index	$CTVI=\left(\frac{NDVI+0.5}{ABS(NDVI+0.5}\right)*{\left(ABS\left(NDVI+0.5\right)\right)}^{0.5}$	Biomass and vegetation vigor	(Perry and Lautenschlager, 1984)
10	NDVIGreen	Normalized Difference Vegetation Index Green	$NDVIGreen=\frac{\left(NIR-REDEDGE\right)}{\left(NIR+GREEN\right)}$	Chlorophyll content and nitrogen status	(Gamon et al., 1995)
11	LCI	Leaf Chlorophyll Index	$LCI=\frac{\left(NIR-REDEDGE\right)}{\left(NIR+RED\right)}$	Chlorophyll content	(Datt, 1999)
12	CIgreen	Chlorophyll Index	$CIgreen=\left(\frac{NIR}{GREEN}\right)-1$	Chlorophyll content and nitrogen status	(Ahamed et al., 2011)
13	CIrededge	Chlorophyll Index Red Edge	$CIrededge=\left(\frac{NIR}{REDEDGE}\right)-1$	Chlorophyll content and photosynthetic activity	(Wu et al., 2009)
14	CCCI	Canopy Chlorophyll Content Index	$CCCI=\frac{\frac{\left(NIR\;-\;REDEDGE\right)}{\left(NIR\;+\;EDEDGE\right)}}{\frac{\left(NIR-RED\right)}{\left(NIR\;+\;RED\right)}}$	Chlorophyll content and nitrogen status	(D. M. El-Shikha et al., 2008)
15	Chlred-edge	Chlorophyll Red-edge	$ChRed\;Edge={\left(\frac{NIR}{REDEDGE}\right)}^{-1}$	Chlorophyll content	(Gitelson et al., 2006)
16	CVI	Chlorophyll Vegetation Index	$CVI=NIR*\left(\frac{RED}{GREEN^{2}}\right)$	Chlorophyll content and leaf area	(Vincini et al., 2008)
17	GVI	Green Vegetation Index	$GVI=\frac{NIR}{GREEN}$	Biomass and green vigor	(Tucker, 1979)
18	NGRD	Normalized Green-Red Difference Index	$NGRD=\frac{GREEN-RED}{GREEN+RED}$	Water stress, pigment ratios, and senescence	(Zarco-Tejada et al., 2001)
19	RG	Red-Green Ratio	$RG=\frac{RED}{GREEN}$	Senescence and shifts in pigment ratios	(Gitelson et al., 2002)
20	GRE	Green Excess Index	GRE=GREEN∗REDEDGE	Biomass and vegetation vigor	(Woebbecke et al., 1995)
21	GBNDVI	Green-Blue Normalized Difference Vegetation Index	$GBNDVI=\left(\frac{NIR-\left(GREEN+BLUE\right)}{NIR+\left(GREEN+BLUE\right)}\right)$	Biomass and leaf structural changes	(Wang et al., 2007)
22	GRNDVI	Green-Red Normalized Difference Vegetation Index	$GRNDVI=\left(\frac{NIR-\left(GREEN+RED\right)}{NIR+\left(GREEN+RED\right)}\right)$	Biomass and chlorophyll status	(Main et al., 2011)
23	HIV	Health Index	$HIV=\left(\frac{GREEN-REDEDGE}{GREEN+REDEDGE}\right)-\left(0.5*REDEDGE\right)$	Water stress and vegetation health status	(Mahlein et al., 2013)
24	VARI	Visible Atmospherically Resistant Index	$VARI=\frac{GREEN-RED}{GREEN+RED-BLUE}$	Biomass and canopy coverage	(Gitelson and Merzlyak, 1994)
25	SIPI	Structure Insensitive Pigment Index	$SIPI=\frac{NIR-BLUE}{NIR-RED}$	Water stress and carotenoid/chlorophyll ratio	(Pu et al., 2008)
26	ARVI	Atmospherically Resistant Vegetation Index	$ARVI=\frac{NIR-RED-y\left(RED-BLUE\right)}{NIR+RED-y\left(RED-BLUE\right)}$	Biomass and greenness	(Gitelson, 2004)
27	RTVI	Red-edge Triangular Vegetation Index	RTVI=(100(NIR−REDEDGE)−10(NIR−GREEN)	Biomass and leaf area	(Haboudane, 2004)
28	EVI	Enhanced Vegetation Index	$EVI=2.5\frac{NIR-RED}{\left(NIR+6*RED-7.5*BLUE\right)+1}$	Biomass and vegetation density	(Huete et al., 2002)
29	MEVI	Modified Enhanced Vegetation Index	$MEVI=2.5\frac{NIR-REDEDGE}{\left(NIR+6*REDEDGE-7.5*BLUE\right)+1}$	Biomass and vegetation vigor	(Huete et al., 2002)
30	NPCI	Normalized Pigment Chlorophyll Index	$NPCI=\frac{RED-BLUE}{RED+BLUE}$	Water stress and total pigment ratios	(Peñuelas et al., 1993)
31	PSRI	Plant Senescence Reflectance Index	$PSRI=\frac{RED-GREEN}{NIR}$	Senescence and carotenoid/chlorophyll ratio	(Merzlyak et al., 1999)
32	TCVI	Transformed Chlorophyll Vegetation Index	$TCVI=1.4\frac{\left(\left(2*RED\right)-\left(2*BLUE\right)\right)}{\left(\left(2*RED\right)-GREEN\left(2*BLUE\right)-0.4\right)}$	Chlorophyll content	(Haboudane, 2004)
33	GDVI	Green Difference Vegetation Index	GDVI=NIR−GREEN	Biomass and vegetation vigor	(Tucker et al., 1979)
34	NLI	Non-Linear Index	$NLI=\frac{(NIR^{2})-RED}{\left(NIR^{2}\right)+RED}$	Biomass and vegetation cover	(Goel and Qin, 1994)
35	TCAVI	Transformed Canopy Vegetation Index	$TCAVI=3\frac{\left(REDEDGE-RED-0.2\right)\left(REDEDGE-GREEN\right)*REDEDGE}{RED}$	Biomass and canopy structure	(Haboudane, 2004)

BLUE: blue spectral band 450 nm, GREEN: green spectral band 560 nm, RED: red spectral band 650 nm, REDEDGE: red edge spectral band 730 nm, NIR: near infrared spectral band 840 nm, CC: Canopy cover in %.

### 2.8 Spectral signatures

The reflectance of the vegetation measured with a spectroradiometer was carried out using the AvaSpec-ULS2048CL-EVO (Avantes, Apeldoorn, Netherlands) at a spectral range of 450 nm to 965 nm, the measurement time was between 8:00 a.m. to 10:00 a.m. and from 2:00 p.m. to 4:00 p.m. The readings were managed by the AvaSoft v.8.15.0.0 software, on a laptop computer that was powered through a USB 2.0 port with a 5.83 cm viewing radius, height 30 cm above the nadir and 11° angle, the readings were taken at 52, 66, 78, 91 and 105 DAS, similar to the diagram in **Figure 2** and following the methodology of (Chávez et al., 2010), in order to characterize spectral signatures of different genotypes.

### 2.9 Data analysis

The study was conducted using a randomized complete block design (RCBD) with two irrigation treatments. Data were recorded in a field notebook across 100 experimental units, and statistical analyses were performed using R v.4.4.1 (R Core Team, n.d.) and RStudio v.2024.04.2-764 (RStudio Team, n.d.). First, an analysis of variance (ANOVA) was conducted to determine the significance of the factors under study: genotype, irrigation treatment, and their interaction. To identify specific differences among means, the Waller-Duncan test was applied at a significance level of α=0.05, due to its suitability for multiple mean comparisons involving a large number of treatments. This *post-hoc* test facilitated detailed comparison of means between treatments and genotypes. Subsequently, multiple correlation analyses were performed using Pearson’s correlation coefficient (r) to examine the interrelation of agromorphological and physiological variables. Subsequently, the ggplot2 (Wickham et al., 2024) and agricolae (Felipe de Mendiburu, 2006) packages in R were used to carry out descriptive statistics and determine the relationships between the evaluated variables. In addition, a principal component analysis (PCA) was performed to discriminate the evaluation dates and vegetation indexes. For the multivariate analysis, R’s factoextra (Kassambara and Mundt, 2020) and FactoMiner (Husson et al., 2024) packages were used.

## 3 Results

### 3.1 Agromorphological traits

Deficit irrigation significantly (p< 0.05) reduced most agromorphological traits across genotypes, although the magnitude of reduction varied considerably (**Tables 6** and **7**). For plant height (PH), the tallest genotypes under deficit irrigation (DI) were ACC_43 (108.6 cm), ACC_44 (100.1 cm), and ACC_20 (100.0 cm), with reductions of 23.8%, 13.4%, and 17.6%, respectively, compared to normal irrigation (NI). However, B_JULY, SALCEDO, and ACC_44 showed smaller height reductions under DI, with decreases of 9.5%, 11.4%, and 13.4%, respectively. Specific leaf area (SLA), which reflects leaf thickness, was lower under DI in SALCEDO (162.6 cm^2^ g^-1^), ACC_20 (172.9 cm^2^ g^-1^), and ACC_44 (173.6 cm^2^ g^-1^), representing reductions of 14.7%, 1.8%, and 7.1%, respectively, compared to NI. In contrast, B_JULY, ACC_20, and ACC_23 exhibited the smallest SLA reductions under DI, with decreases of 0.0%, 1.8%, and 3.4%, respectively.

**Table 6:** Agromorphological variables of 10 quinoa genotypes; PH, SLA, PL, PD, and TGW.

Genotype	PH (cm)					SLA (cm^2^g^-1^)					PL (cm)					PD (cm)					TGW (g)				
	NI		DI			NI		DI			NI		DI			NI		DI			NI		DI		
SALCEDO	108.48	_cd_	96.09	_bc_	*ns*	190.56	_ab_	162.62	_b_	*ns*	46.73	_ab_	27.33	_bc_	*	15.57	_cd_	8.73	_a_	*	2.77	_b_	2.08	_b_	*
B_JULY	90.43	_e_	81.88	_d_	*ns*	173.33	_b_	173.73	_ab_	*ns*	42.50	_b_	25.37	_c_	*	23.20	_a_	8.30	_a_	*	1.12	_g_	0.88	_e_	*ns*
ACC_50	112.22	_bcd_	87.52	_cd_	*ns*	243.35	_a_	196.67	_a_	*	42.17	_b_	30.07	_abc_	*	12.47	_d_	9.53	_a_	*ns*	2.94	_ab_	2.60	_a_	*ns*
ACC_49	98.06	_de_	79.16	_d_	*	201.46	_ab_	176.85	_ab_	*ns*	43.77	_b_	27.57	_bc_	*	16.83	_bcd_	7.87	_a_	*	3.03	_a_	2.79	_a_	*ns*
ACC_44	115.57	_bc_	100.09	_ab_	*	186.79	_b_	173.61	_ab_	*ns*	48.80	_ab_	29.67	_abc_	*	19.20	_abc_	10.67	_a_	*	2.01	_de_	1.74	_c_	*
ACC_43	142.37	_a_	108.55	_a_	*	186.15	_b_	177.55	_ab_	*ns*	51.80	_a_	30.90	_ab_	*	17.43	_bc_	10.67	_a_	*	2.29	_c_	1.72	_c_	*
ACC_32	98.76	_de_	79.71	_d_	*	196.85	_ab_	174.72	_ab_	*ns*	47.93	_ab_	27.87	_bc_	*	19.37	_abc_	9.77	_a_	*ns*	1.91	_ef_	1.68	_c_	*ns*
ACC_23	120.44	_bc_	97.44	_abc_	*	201.41	_ab_	194.66	_a_	*ns*	49.37	_ab_	29.43	_abc_	*	21.17	_ab_	8.37	_a_	*	2.25	_cd_	1.88	_bc_	*ns*
ACC_20	121.47	_bc_	100.04	_ab_	*ns*	176.04	_b_	172.91	_ab_	*ns*	52.22	_a_	34.17	_a_	*	19.53	_abc_	8.00	_a_	*	1.69	_f_	1.24	_d_	*ns*
ACC_06	124.06	_b_	95.87	_bc_	*	187.24	_b_	180.38	_ab_	*ns*	51.83	_a_	30.80	_ab_	*	17.70	_bc_	8.37	_a_	*	0.92	_g_	0.94	_de_	*ns*
Promedio	113.18		92.63		*	193.63		179.06		*	47.12		29.57		*	18.25		9.03		*	2.09		1.76		*

PH, plant height; SLA, specific leaf area; PL, panicle length; PD, panicle diameter; TGW, thousand grain weight; NI, normal irrigation; DI, deficit irrigation. Different letters indicate significant differences among genotypes at test (p< 0.05). Data are presented as means of five replicates (n = 5). Differences among treatments, * significant at 0.05 level according to F-test; ns: is non-significant.

**Table 7:** Agromorphological variables of 10 quinoa genotypes under NI and DI; GD, GT, Y, and HI.

Genotype	GD (mm)					GT (mm)					Y (t ha^-1^)					HI				
	NI		DI			NI		DI			NI		DI			NI		DI		
SALCEDO	2.10	_b_	1.97	_b_	*	1.14	_ab_	1.02	_a_	*	1.05	_cd_	0.62	_de_	*ns*	0.14	_cdef_	0.09	_cd_	*
B_JULY	1.69	_f_	1.62	_ef_	*ns*	0.83	_f_	0.82	_c_	*ns*	0.18	_e_	0.17	_f_	*ns*	0.03	_f_	0.05	_d_	*ns*
ACC_50	2.21	_a_	2.12	_a_	*	1.17	_a_	1.04	_a_	*	1.97	_ab_	1.19	_b_	*	0.44	_a_	0.29	_a_	*ns*
ACC_49	2.13	_b_	2.08	_a_	*ns*	1.07	_bc_	1.03	_a_	*ns*	2.55	_a_	1.51	_a_	*	0.35	_ab_	0.29	_a_	*ns*
ACC_44	1.88	_d_	1.84	_d_	*ns*	1.04	_cd_	0.94	_b_	*	1.29	_c_	0.53	_de_	*	0.15	_cdef_	0.11	_c_	*ns*
ACC_43	2.00	_c_	1.87	_cd_	**	0.98	_de_	0.86	_c_	*	1.48	_bc_	0.62	_de_	**	0.17	_cde_	0.08	_cd_	*
ACC_32	1.81	_e_	1.81	_d_	*ns*	0.99	_de_	0.94	_b_	*ns*	1.34	_bc_	0.91	_bc_	*ns*	0.25	_bc_	0.21	_b_	*ns*
ACC_23	1.93	_d_	1.91	_c_	*ns*	1.04	_cd_	0.97	_ab_	*ns*	1.43	_bc_	0.80	_cd_	*ns*	0.20	_cd_	0.12	_c_	*ns*
ACC_20	1.81	_e_	1.67	_e_	**	0.95	_e_	0.84	_c_	*ns*	0.66	_de_	0.38	_ef_	*ns*	0.09	_def_	0.07	_cd_	*ns*
ACC_06	1.68	_f_	1.60	_f_	***	0.81	_f_	0.74	_d_	*	0.41	_e_	0.37	_ef_	*ns*	0.07	_ef_	0.07	_cd_	*ns*
Promedio	1.92		1.85		*	1.00		0.92		*	1.24		0.71		***	0.19		0.14		*

GD, Grain diameter; GT, grain thickness; Y, Yield; HI, harvest index; NI, normal irrigation; DI, deficit irrigation. Different letters indicate significant differences among genotypes at test (p< 0.05). Data are presented as means of five replicates (n = 5). Differences among treatments, * significant at 0.05 level according to F-test; ns: is non-significant.

For panicle length (PL), the genotypes with the largest panicles under NI were ACC_20 (52.2 cm), ACC_43 (51.8 cm), and ACC_06 (51.8 cm), with reductions of 34.6%, 40.3%, and 40.6%, respectively, under DI. However, ACC_50 and ACC_20 showed smaller reductions in PL, by 28.7% and 34.6%, respectively, under DI. For panicle diameter (PD), the highest values under DI were recorded in ACC_44 (10.7 cm), ACC_43 (10.7 cm), and ACC_32 (9.8 cm), although these values represented reductions of 44.4%, 38.8%, and 49.6%, respectively, compared to NI. In contrast, ACC_50 showed a smaller reduction in PD (23.6%).

For thousand grain weight (TGW), the highest values were recorded in ACC_49 (3.03 g), ACC_50 (2.94 g), and SALCEDO (2.77 g), with reductions under DI of 7.9%, 11.6%, and 24.9%, respectively. Grain diameter (GD) was highest in ACC_50 (2.2 mm), ACC_49 (2.1 mm), and SALCEDO (2.1 mm) under NI, with reductions of 4.1%, 2.3%, and 6.2%, respectively, under DI. The smallest reductions in GD were observed in ACC_32 and ACC_23, with reductions of 0.0% and 1.0%, respectively. Regarding grain thickness (GT), ACC_50 (1.2 mm), ACC_49 (1.1 mm), and SALCEDO (1.1 mm) showed the highest values under NI, with reductions of 11.1%, 3.7%, and 10.5%, respectively, under DI. However, B_JULY showed the smallest reduction in GT (1.2%).

Yield (Y) was the most severely impacted trait, with an average reduction of 42.6%. Genotypes ACC_49 and ACC_50 were the highest yielding under both NI (2.55 and 1.97 t ha^-1^) and DI (1.51 and 1.19 t ha^-1^), while B_JULY and ACC_06 showed remarkable stability with the smallest yield reductions (5.6% and 9.8%, respectively). Similarly, for harvest index (HI), which reflects the conversion of biomass into harvestable grain, ACC_50 (0.44), ACC_49 (0.35), and ACC_32 (0.25) showed the highest values under NI, while HI was reduced by 34.1%, 17.1%, and 16.0%, respectively, under DI. In contrast, B_JULY and ACC_06 maintained stable HI values under DI.

### 3.2 Physiological traits

In **Table 8**, relative chlorophyll content (SPAD), which reflects the relative level of chlorophyll in the leaves, was highest under deficit irrigation (DI) in ACC_43 (59.6), ACC_20 (59.0), and ACC_44 (58.3), with increases of 20.2%, 16.6%, and 20.2%, respectively, compared with normal irrigation (NI). The lowest SPAD increases under DI were observed in B_JULY, ACC_06, and ACC_23, with increases of 5.6%, 9.5%, and 12.1%, respectively. This increase in SPAD under DI may reflect a concentration effect associated with reduced leaf expansion and lower specific leaf area, which can result in thicker leaves and a higher relative chlorophyll concentration per unit leaf area.

**Table 8:** Physiological variables of 10 NI and DI quinoa genotypes; SPAD index, RWC, DM, and SD.

Genotype	SPAD					RWC (%)					DM (g planta^-1^)					SD (Stomata mm^-2^)				
	NI		DI			NI		DI			NI		DI			NI		DI		
SALCEDO	44.9	_def_	51.9	_cde_	*	80.6	_a_	77.8	_abc_	*ns*	27.8	_a_	22.5	_ab_	*ns*	109	_bc_	128	_cd_	*ns*
B_JULY	46.6	_bcde_	49.2	_de_	*ns*	85.9	_a_	82.3	_ab_	*ns*	27.6	_a_	14.9	_b_	*	140	_a_	144	_abc_	*ns*
ACC_50	40.6	_f_	48.1	_e_	*	82.2	_a_	79.0	_abc_	*ns*	31.6	_a_	27.2	_ab_	*ns*	98	_c_	107	_d_	*ns*
ACC_49	43.6	_ef_	49.4	_de_	*	84.5	_a_	78.8	_abc_	*ns*	41.6	_a_	18.7	_ab_	*ns*	103	_c_	122	_cd_	*ns*
ACC_44	48.5	_abcd_	58.3	_ab_	*	84.8	_a_	73.5	_c_	*	28.4	_a_	16.0	_b_	*ns*	119	_abc_	170	_ab_	*
ACC_43	49.6	_abc_	59.6	_a_	*	77.5	_a_	73.9	_c_	*ns*	35.7	_a_	17.8	_b_	*	141	_a_	172	_a_	*ns*
ACC_32	45.4	_cde_	56.1	_abc_	*	82.3	_a_	75.5	_bc_	*ns*	27.9	_a_	18.2	_b_	*ns*	119	_abc_	135	_bcd_	*ns*
ACC_23	48.0	_abcde_	53.8	_bcd_	*ns*	88.7	_a_	78.4	_abc_	*	46.7	_a_	32.4	_a_	*	113	_abc_	127	_cd_	*ns*
ACC_20	50.6	_ab_	59.0	_ab_	*	86.2	_a_	80.8	_abc_	*	28.7	_a_	24.1	_ab_	*ns*	133	_ab_	140	_abcd_	*ns*
ACC_06	51.5	_a_	56.4	_abc_	*ns*	88.1	_a_	83.6	_a_	*ns*	35.1	_a_	20.0	_ab_	*	134	_ab_	144	_abc_	*ns*
Promedio	46.9		54.2		*	84.1		78.3		*	33.1		21.2		*	121		139		**

SPAD, relative chlorophyll content; RWC, relative water content; DM, Dry matter weight; SD, stomatal density; NI, normal irrigation; DI, deficit irrigation. Different letters indicate significant differences among genotypes at test (p< 0.05). Data are presented as means of five replicates (n = 5). Differences among treatments, * significant at 0.05 level according to F-test; ns: is non-significant.

For relative water content (RWC), the highest values under NI were recorded in ACC_23 (88.7%), ACC_06 (88.1%), and ACC_20 (86.2%), which decreased by 11.6%, 5.1%, and 6.3%, respectively, under DI. The smallest reductions in RWC under DI were observed in SALCEDO (3.5%) and ACC_50 (3.9%). For dry matter (DM), the highest values under NI were recorded in ACC_23 (46.7 g plant^-1^), ACC_49 (41.6 g plant^-1^), and ACC_50 (31.6 g plant^-1^), with reductions of 30.6%, 55.0%, and 13.9%, respectively, under DI. Meanwhile, the genotypes with the smallest reductions in DM under DI were ACC_50 (13.9%) and ACC_20 (16.0%).

For stomatal density (SD), the highest number of stomata per mm^2^ under DI was observed in ACC_43 (172), ACC_44 (170), and ACC_06 (144), which increased SD relative to NI by 22.0%, 42.9%, and 7.5%, respectively. However, the genotypes that showed the smallest increases in SD due to DI were B_JULY (2.9%), ACC_20 (5.3%), and ACC_06 (7.5%).

**Table 9:** Response of genotypes to water stress evaluated using tolerance indices.

Genotype	GMP	HM	STI	TOL	SSI	SI	SNPI
SALCEDO	0.81	1.87	0.43	0.43	0.96	0.41	1.26
B_JULY	0.17	0.51	0.02	0.01	0.11	0.05	0.46
ACC_50	1.53	3.57	1.53	0.78	0.93	0.40	3.36
ACC_49	1.96	4.53	2.52	1.04	0.96	0.41	4.77
ACC_44	0.83	1.60	0.45	0.76	1.38	0.59	0.94
ACC_43	0.96	1.85	0.60	0.86	1.37	0.58	1.17
ACC_32	1.11	2.74	0.80	0.43	0.75	0.32	2.43
ACC_23	1.07	2.41	0.75	0.62	1.02	0.44	1.82
ACC_20	0.50	1.14	0.16	0.28	1.01	0.43	0.59
ACC_06	0.39	1.10	0.10	0.05	0.27	0.11	0.96
Average	0.94	2.13	0.57	0.53	1.00	0.43	1.52

GMP, Geometric Mean Productivity; HM, Harmonic Mean; STI, Stress Tolerance Index; TOL, Stress Tolerance Index; SSI, Stress Susceptibility Index; SI, Stress Intensity; SNPI, Stress and Non-Stress Production Index. Dark red shading represents values closest to 0, whereas dark green shading represents values closest to 5.

### 3.3 Analysis of tolerance selection indices

The drought tolerance indices of the evaluated quinoa genotypes are presented in **Table 9**. In terms of tolerance selection, ACC_49 and ACC_50 genotypes performed better because they combined high yield with good stability under ID conditions. These genotypes led in yield, achieving the highest geometric mean (GMP) values of 1.96 and 1.53 and harmonic mean (HM) values of 4.53 and 3.57, respectively. However, genotypes B_JULY and ACC_06 gave the lowest yields in both NI and DI, representing minimum values for the same yield parameters (GMP< 0.40).

For the stress susceptibility index (SSI), which measures the effect of deficit irrigation on the genotype, the highest values were seen in ACC_44 (1.38) and ACC_43 (1.37), being considered the most susceptible, because they suffered the greatest reduction in their DI performance. Likewise, the lowest values of SSI and tolerance (TOL) occurred in genotype B_JULY (0.11 and 0.05, respectively); however, this does not indicate real tolerance, but rather a very low yield, unlike ACC_32, which combined genuine low susceptibility (SSI = 0.75) with high productivity (HM = 2.74).

### 3.4 Water use efficiency of genotypes

**Figure 3:**
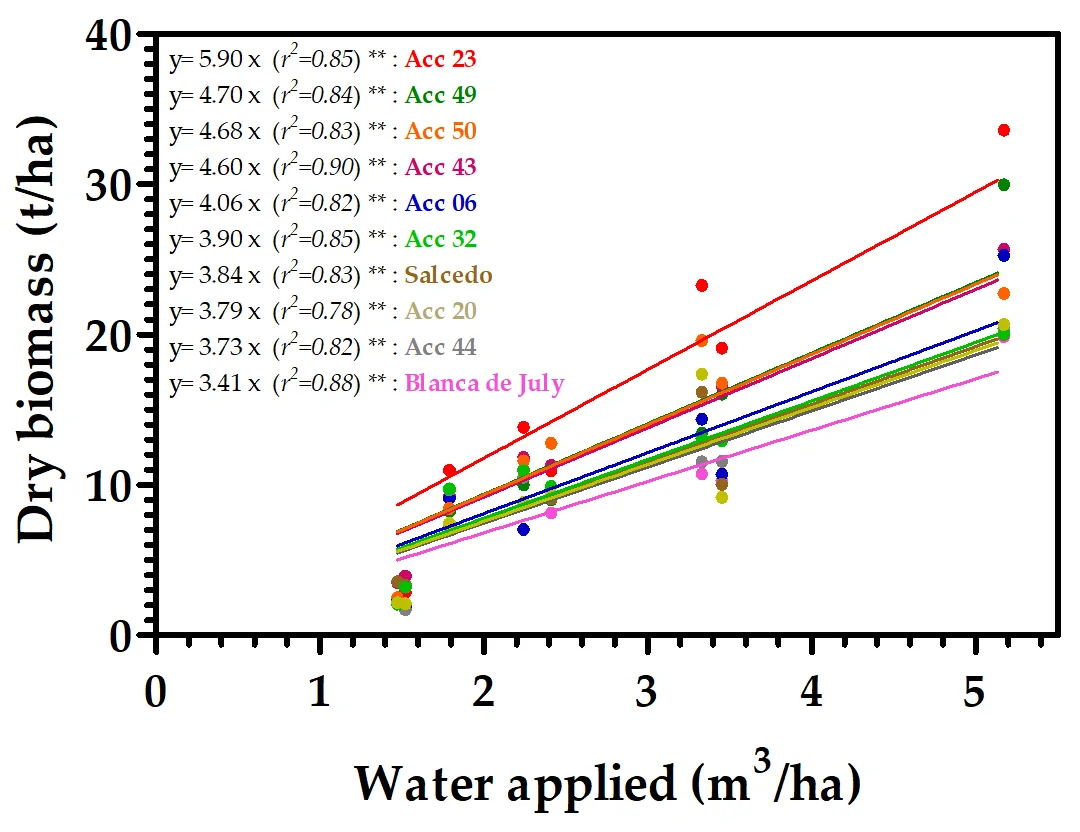
Water use efficiency based on the ratio between dry matter and water applied.

With respect to the production of dry biomass as a function of water applied (**Figure 3**), a highly significant positive linear response was observed in all genotypes (R^2^ = 0.78). In this analysis, genotype ACC_23 showed the highest water use efficiency (WUE) for biomass production (5.90 g kg^-1^), followed by ACC_49 (4.70 g kg^-1^) and ACC_50 (4.68 g kg^-1^). In contrast, B_JULY exhibited the lowest water use efficiency (3.41 g kg^-1^), indicating limited responsiveness to water availability. However, the high WUE observed in ACC_23 did not translate into the highest grain yield, suggesting that greater biomass production efficiency does not necessarily imply superior reproductive performance under deficit irrigation conditions. Likewise, high water use efficiency for biomass did not necessarily translate into higher grain yield. For instance, despite its high efficiency, ACC_23 did not rank among the top-yielding genotypes, whereas ACC_49 and ACC_50 combined relatively high water use efficiency with superior grain yield. This suggests that biomass production efficiency alone is not sufficient to predict yield performance and highlights the importance of biomass partitioning in breeding programs under water-limited conditions. Likewise, the coefficients of determination (R^2^) ranged between 0.78 and 0.90, confirming that the linear model adequately explained biomass variation in response to the applied irrigation gradient.

### 3.5 Sensitivity of VIs to deficit irrigation

As a result of the sensitivity of the LUs to the NI and DI irrigation treatments, the statistical differences found in the student’s t-test were classified, according to the frequency of the number of these differences (**Table 10**), for each p-value (*:0.05, **:0.01, ***:0.001). Vegetation indices HIV, NGRD, RG and PSRI VIs topped the ranking, showing the highest detection capacity (high sensitivity, p<0.01) for most of the dates and genotypes evaluated.

**Table 10:** Order of IVs based on probability value.

Ranking^1^ of vegetation indexes (IVs) for detecting water stress											
N°	VIs	Sig.	*	**	***	N°	VIs	Sig.	*	**	***
1	HIV	31	10	11	10	19	EVI	28	8	16	4
2	NGRD	32	10	14	8	20	CIgreen	20	8	8	4
3	RG	32	11	13	8	21	GVI	20	8	8	4
4	PSRI	26	10	8	8	22	CVI	10	2	4	4
5	VARI	31	14	11	6	23	SAVI-CC	27	9	15	3
6	NDVI	29	12	11	6	24	AVI	27	9	15	3
7	TTI	29	12	11	6	25	GDVI	24	9	12	3
8	IPVI	29	12	11	6	26	TCAVI	13	4	6	3
9	CTVI	29	12	11	6	27	TCVI	10	5	2	3
10	Chlred-edge	29	12	11	6	28	SIPI	13	6	5	2
11	NRVI	28	12	10	6	29	GRE	10	4	4	2
12	GBNDVI	26	9	11	6	30	RTVI	9	7	2	0
13	GRNDVI	24	10	8	6	31	MEVI	8	6	2	0
14	RVI	28	12	11	5	32	LCI	7	5	2	0
15	ARVI	25	9	11	5	33	NPCI	6	1	5	0
16	NDVIGreen	21	9	7	5	34	CCCI	5	5	0	0
17	NLI	29	11	14	4	35	CIrededge	3	2	1	0
18	SAVI-0.5	28	9	15	4						

^1^ The order of the following table is based on the probability value when the differences are highly significant.

p-value at significance level; *:0.05, **:0.01, ***:0.001, for the number of times it identifies significant differences (Sig.) and counted in the table.

HIV, Health Index; NGRD, Normalized Green-Red Difference Index; RG, Red-Green Ratio; PSRI, Plant Senescence Reflectance Index; VARI, Visible Atmospherically Resistant Index; NDVI, Normalized Difference Vegetation Index; TTI, Transformed Vegetation Index; IPVI, Infrared Percentage Vegetation Index; CTVI, Corrected Transformed Vegetation Index; Chlred-edge, Chlorophyll Red-edge; NRVI, Normalized Ratio Vegetation Index; GBNDVI, Green-Blue Normalized Difference Vegetation Index; GRNDVI, Green-Red Normalized Difference Vegetation Index; RVI, Ratio Vegetation Index; ARVI, Atmospherically Resistant Vegetation Index; NDVIGreen, Normalized Difference Vegetation Index Green; NLI, Non-Linear Index; SAVI-0.5, Soil-Adjusted Vegetation Index with L factor of 0.5; EVI, Enhanced Vegetation Index; CIgreen, Chlorophyll Index; GVI, Green Vegetation Index; CVI, Chlorophyll Vegetation Index; SAVI-CC, Soil-Adjusted Vegetation Index with Canopy Cover; AVI, Advanced Vegetation Index; GDVI, Green Difference Vegetation Index; TCAVI, Transformed Canopy Vegetation Index; TCVI, Transformed Chlorophyll Vegetation Index; SIPI, Structure Insensitive Pigment Index; GRE, Green Excess Index; RTVI, Red-edge Triangular Vegetation Index; MEVI, Modified Enhanced Vegetation Index; LCI, Leaf Chlorophyll Index; NPCI, Normalized Pigment Chlorophyll Index; CCCI, Canopy Chlorophyll Content Index; CIrededge, Chlorophyll Index Red Edge.

### 3.6 Spectral signatures

**Figure 4:**
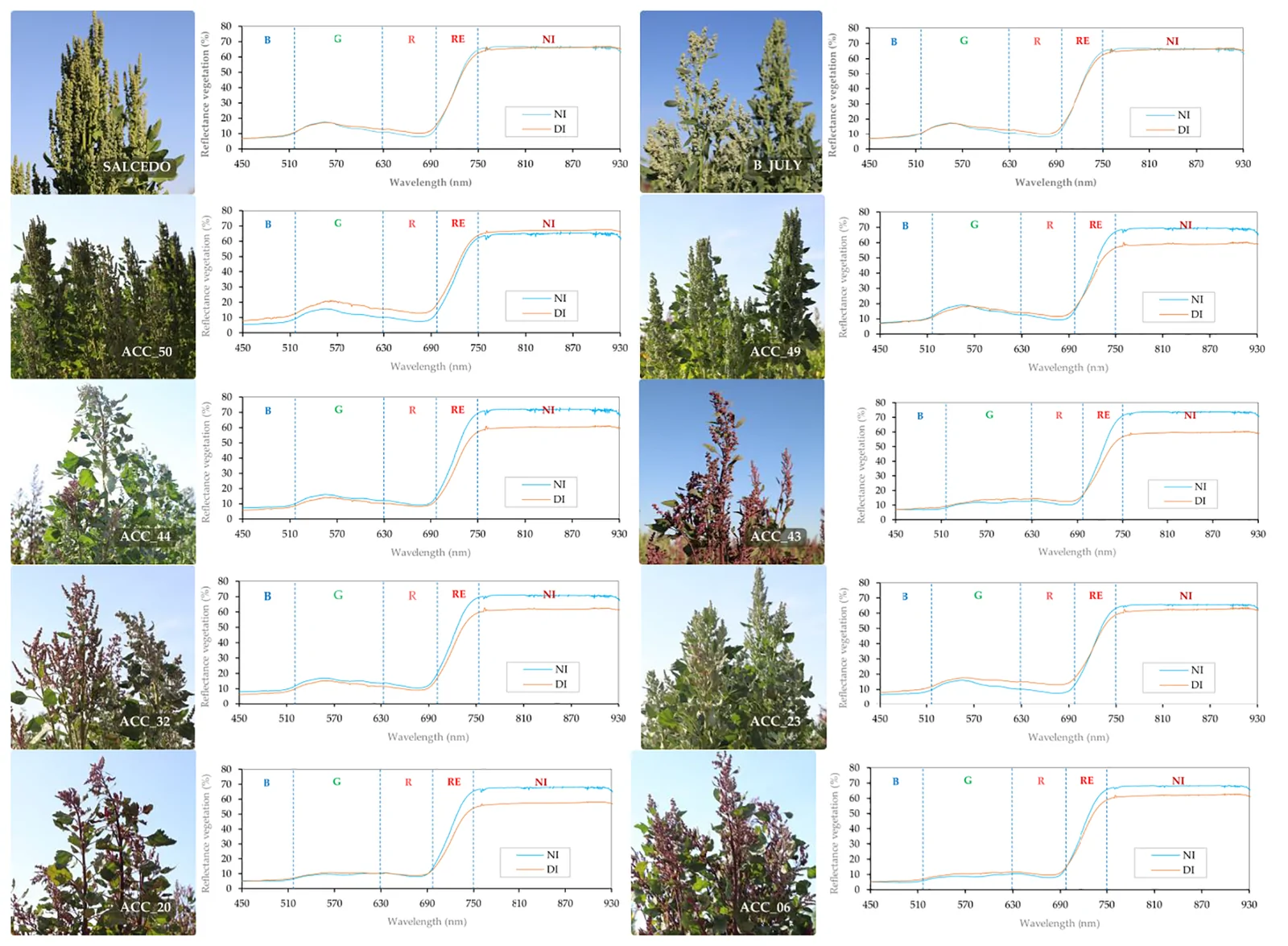
Spectral signatures acquired from genotypes due to treatment. B (Blue spectral range): 450 nm-520 nm; G (Green spectral range): 520 nm-600 nm; R (Red spectral range): 630 nm-690 nm; RE (Red Edge spectral range): 690 nm-740 nm; NIR (Near-Infrared spectral range): 750 nm-900 nm; NI, normal irrigation; DI, deficit irrigation.

In **Figure 4**, the spectral signatures of quinoa genotypes under different irrigation treatments showed notable differences, particularly in the near-infrared (NIR: 750–930 nm) region. The SALCEDO and B_JULY genotypes exhibited high NIR reflectance and strong absorption in the blue (450–510 nm) and red (630–690 nm) regions, with similar spectral responses under both irrigation conditions. The maintenance of high NIR reflectance under deficit irrigation (DI) suggests better preservation of leaf internal structure and cellular turgor, which are closely associated with light scattering within the mesophyll.

In contrast, genotypes ACC_43, ACC_44, ACC_49, and ACC_20 showed reduced NIR reflectance under DI, indicating changes in leaf structural integrity that were likely related to decreased tissue hydration. Genotype ACC_50 exhibited increased reflectance in the visible range, which may be associated with reduced chlorophyll content. Additionally, variations observed in the red-edge region indicate changes in chlorophyll concentration and photosynthetic activity under water deficit conditions.

### 3.7 Statistical correlation analysis of evaluated variables

**Figure 5:**
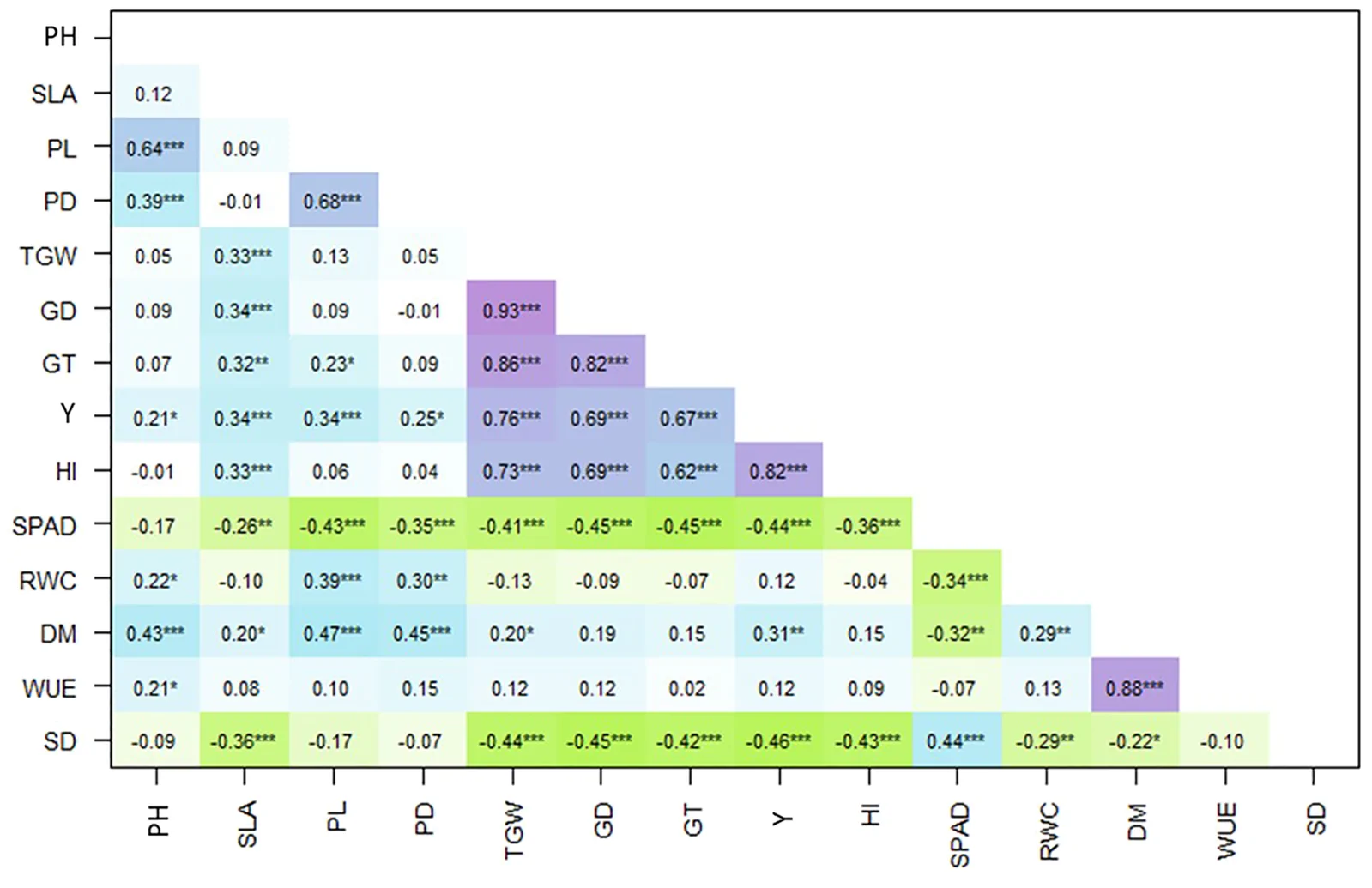
Pearson’s r correlation between agromorphological and physiological variables of 10 quinoa genotypes under normal and deficit irrigation treatments. PH, plant height; SLA, specific leaf area; PL, panicle length; PD, panicle diameter; TGW, thousand grain weight; GD, Grain diameter; GT, grain thickness; Y, Yield; HI, harvest index; SPAD, relative chlorophyll content; RWC, relative water content; DM, Dry matter weight; WUE, water use efficiency; SD, stomatal density.

In **Figure 5**, the correlation of physiological and agromorphological variables revealed the existence of significant relationships, highlighting highly significant positive correlations between TGW versus GD (r=0.93***), WUE versus DM (r=0.88***), TGW versus TD (r=0.86***), Y versus HI (r=0.82***), Y versus TGW (r = 0.76***), and TGW versus HI (r=0.73). On the other hand, moderate correlations were observed between PL versus PD (r = 0.68***), PH versus PL (r=0.64***), and SPAD versus GD (r=-0.56***). Correlations were low for SPAD versus GT (r=-0.45***), GD versus SD (r=-0.45***), and SD versus Y (r=-0.46***).

The principal component analysis (PCA) shows a clear distribution of the 35 vegetation indices evaluated. Since the indices formulated in NIR/RED are related to biomass and photosynthetic activity (NDVI, RVI, NRVI, TTI, EVI, MEVI and ARVI), they were projected towards the upper right quadrant, indicating an association with positive PC1 and positive PC2. Similarly, indices evaluating chlorophyll and leaf pigments (LCI, CIgreen, CIrededge, CCCI, Chlred-edge, CVI, TCVI, and NPCI) showed a similar orientation, possibly having a high correlation between chlorophyll indices and biomass indices. On the other hand, the indices associated with the soil adjustment were projected towards the lower left quadrant (SIPI) and lower right quadrant (NPCI, RG, PSRI). An association was also found between the VIs TCAVI, VARI, NGRD and GRE, which are located in the upper left quadrant and share the primary band of green.

**Figure 6:**
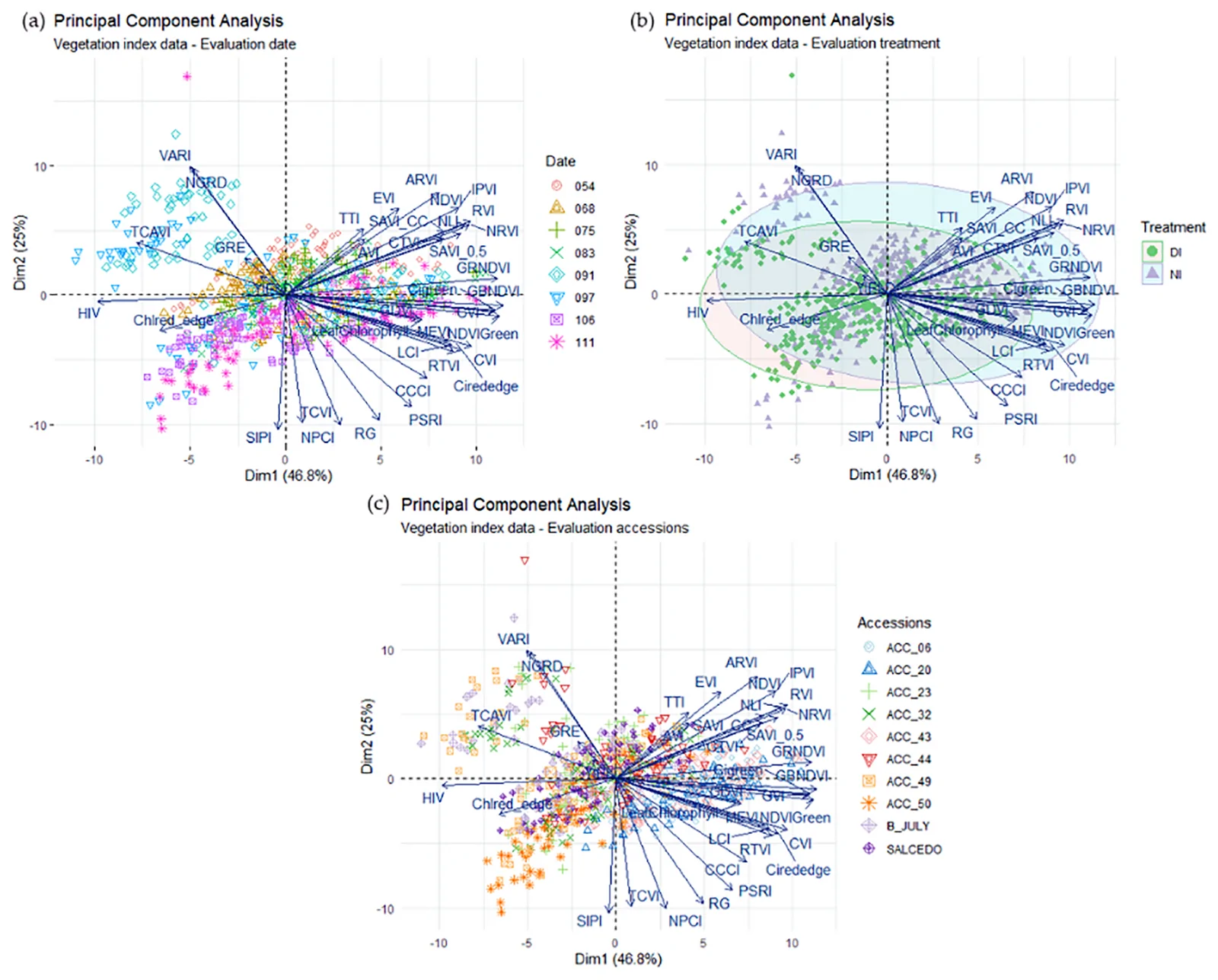
Biplot graph of the PCA showing the multivariate analysis of the principal components of the 35 vegetation indexes **(VIs)** in quinoa **(a)** dates evaluated (DAS) after the start of irrigation treatment, **(b)** DI and NI treatments, and **(c)** genotypes. HIV, Health Index; NGRD, Normalized Green-Red Difference Index; RG, Red-Green Ratio; PSRI, Plant Senescence Reflectance Index; VARI, Visible Atmospherically Resistant Index; NDVI, Normalized Difference Vegetation Index; TTI, Transformed Vegetation Index; IPVI, Infrared Percentage Vegetation Index; CTVI, Corrected Transformed Vegetation Index; Chlred-edge, Chlorophyll Red-edge; NRVI, Normalized Ratio Vegetation Index; GBNDVI, Green-Blue Normalized Difference Vegetation Index; GRNDVI, Green-Red Normalized Difference Vegetation Index; RVI, Ratio Vegetation Index; ARVI, Atmospherically Resistant Vegetation Index; NDVIGreen, Normalized Difference Vegetation Index Green; NLI, Non-Linear Index; SAVI-0.5, Soil-Adjusted Vegetation Index with L factor of 0.5; EVI, Enhanced Vegetation Index; CIgreen, Chlorophyll Index; GVI, Green Vegetation Index; CVI, Chlorophyll Vegetation Index; SAVI-CC, Soil-Adjusted Vegetation Index with Canopy Cover; AVI, Advanced Vegetation Index; GDVI, Green Difference Vegetation Index; TCAVI, Transformed Canopy Vegetation Index; TCVI, Transformed Chlorophyll Vegetation Index; SIPI, Structure Insensitive Pigment Index; GRE, Green Excess Index; RTVI, Red-edge Triangular Vegetation Index; MEVI, Modified Enhanced Vegetation Index; LCI, Leaf Chlorophyll Index; NPCI, Normalized Pigment Chlorophyll Index; CCCI, Canopy Chlorophyll Content Index; CIrededge, Chlorophyll Index Red Edge.

In **Figure 6a**, the dates of 54, 64, 68, and 75 DAS showed greater dispersion than the other evaluation dates and were strongly associated with the health index (HIV), indicating higher spectral variability during the early and mid-development stages. As the plants progressed in development (83, 91, and 97 DAS), the observations gradually shifted toward the center and right side of the biplot, with stronger associations with vegetation indices related to biomass and chlorophyll content, such as NDVI, VARI, NSRD, and ARVI. These associations reached their maximum expression at 106 and 111 DAS, corresponding to the stage of maximum vegetative development. Overall, the early to mid-season stages (54–75 DAS) exhibited greater spectral variability, likely reflecting differential initial responses to deficit irrigation, whereas the later stages (106–111 DAS) showed greater convergence as the plants approached maturity, with indices such as NDVI and VARI capturing the final canopy biomass.

In **Figure 6b**, the PCA showed differences in the response of the VIs under DI and NI treatments. The points corresponding to NI exhibited greater dispersion along the first two principal components, whereas DI treatments showed a narrower clustering pattern. Indices such as VARI and NGRD were more closely associated with NI, while Chlred-edge and CIrededge were more related to DI conditions. The wider dispersion of NI points suggests greater spectral variation under well-watered conditions, whereas DI homogenized the canopy response, forcing plants into a more constrained physiological state.

In **Figure 6c**, genotypes ACC_20 and ACC_23 were associated with high-biomass indices in the upper-right quadrant, consistent with their higher dry matter (DM) accumulation and plant height (PH) values reported earlier. In contrast, ACC_50 and B_JULY were positioned away from these biomass-related indicators, corroborating their distinct adaptive strategies, with ACC_50 prioritizing yield stability and B_JULY showing greater structural rigidity under deficit irrigation. The remaining genotypes exhibited intermediate distributions near the origin, while SALCEDO showed a spectral response representative of the overall population behavior.

## 4 Discussion

### 4.1 Agromorphological responses under constant deficit irrigation

The constant application of 50% deficit irrigation induced substantial morphological adjustments across the quinoa germplasm, reflecting structural acclimation to controlled water limitation rather than a sudden stress response (Jacobsen et al., 2003; Saddiq et al., 2021). Reductions in plant height, panicle length, thousand-grain weight, and grain yield were consistent with quinoa’s sensitivity to restricted water availability (Al-Naggar et al., 2017; Khaled et al., 2021).These changes are likely associated with sustained abscisic acid (ABA)-mediated signaling from drying roots, which restricts cell division and elongation in shoots to reduce vegetative expansion and limit transpirational demand under deficit irrigation (Hinojosa et al., 2018; Mirsafi et al., 2024).

However, the magnitude of the response differed markedly among genotypes, revealing contrasting adaptation strategies. ACC_49 and ACC_50 maintained higher yield stability and harvest index than the commercial cultivars, particularly B_JULY and SALCEDO, indicating a more efficient balance between vegetative growth and reproductive partitioning (Lupa-Condo et al., 2024). In ACC_50, the ability to sustain biomass and reproductive structures under deficit irrigation suggests effective osmotic adjustment and maintenance of source-sink function, which likely supported grain filling despite restricted water supply (Lin and Chao, 2021; Ain et al., 2023). This suggests that a key tolerance mechanism in ACC_50 is the maintenance of reproductive structures under sustained deficit irrigation, preserving sink capacity for grain filling under stress conditions. In contrast, B_JULY appeared to follow a more conservative drought-avoidance strategy focused on maintaining tissue hydration and structural stability, although with lower reproductive allocation under restricted irrigation.

ACC_23 showed high vegetative biomass but comparatively lower grain yield, a pattern consistent with a “vigor trap” in which excessive canopy development increases transpirational demand without proportional reproductive return (El-Harty et al., 2023; Sun et al., 2014). The contrasting behavior between late-maturing genotypes such as B_JULY and ACC_06 and the high-yielding but more sensitive ACC_49 highlights a fundamental trade-off between yield stability and productivity potential under deficit irrigation. Late maturity may provide greater physiological flexibility and prolonged maintenance of active tissues under stress, whereas highly productive genotypes may achieve superior yield potential under favorable conditions but exhibit greater sensitivity to sustained water limitation (Choukr-Allah et al., 2016; Garcia-Parra et al., 2019).

Specific leaf area and stomatal density further clarified these differences. The reduction in SLA observed in several genotypes indicates thicker leaves, a trait associated with reduced water loss and higher chlorophyll density per unit area (Lupa-Condo et al., 2024). Meanwhile, the marked increase in stomatal density in ACC_44 suggests a highly plastic anatomical response, possibly aimed at preserving CO2 uptake during brief favorable periods, although this strategy may also increase vulnerability under prolonged water restriction (Issa Ali et al., 2019). Overall, the agromorphological superiority of ACC_49 and ACC_50 under deficit irrigation appears to depend on maintaining reproductive development and harvest index rather than maximizing vegetative vigor (Geerts et al., 2008; Dumschott et al., 2022).

### 4.2 Physiological responses and genotype-specific strategies

Physiological responses under constant deficit irrigation reflected a trade-off between hydration maintenance and biomass accumulation. The preservation of relative water content in ACC_49, ACC_50, and SALCEDO indicates stronger internal water regulation, likely linked to more effective osmotic adjustment through the accumulation of compatible solutes such as proline and soluble sugars (Farooq et al., 2009; Hinojosa et al., 2018; Ain et al., 2023). In contrast, ACC_06 and B_JULY showed stronger declines in relative water content, suggesting a more limited capacity to buffer tissue dehydration (Issa Ali et al., 2019).

SPAD responses provided additional evidence of structural acclimation. The higher SPAD values observed in stressed genotypes likely reflect a concentration effect caused by reduced leaf expansion and leaf thickening rather than a true increase in chlorophyll biosynthesis (Puangbut et al., 2017; Saddiq et al., 2021). This interpretation is supported by the inverse relationship between SPAD and SLA, indicating that higher pigment density per unit area was associated with structurally thicker leaves. In this context, ACC_50 appears to combine stable chlorophyll status with stronger hydration maintenance, suggesting a more balanced physiological response than genotypes that exhibited high SPAD values primarily as a consequence of restricted leaf expansion (Yang et al., 2016).

The integration of SPAD, RWC, biomass accumulation, and stomatal density suggests contrasting drought-adaptation strategies among genotypes. The ability of B_JULY to maintain high RWC with minimal stomatal density variation points to a conservative drought-avoidance strategy associated with stable tissue hydration and reduced anatomical plasticity. Conversely, ACC_44 exhibited a strong increase in stomatal density under deficit irrigation, which may represent an acclimation response aimed at maximizing CO2 uptake during limited periods of stomatal opening, although at a potentially higher risk of water loss under prolonged stress (Issa Ali et al., 2019; Lin and Chao, 2021).

ACC_23 again illustrates the physiological cost of excessive vegetative vigor. Although biomass accumulation remained high, this did not translate into superior reproductive performance, likely because the larger canopy size increased water demand and accelerated the transition to resource limitation during grain filling (El-Harty et al., 2023; Sun et al., 2014). By contrast, ACC_49 and ACC_50 maintained a more favorable balance between tissue hydration, photosynthetic stability, and assimilate partitioning toward reproductive sinks.

### 4.3 Tolerance indices and water use efficiency

Yield-based tolerance indices showed that GMP and HM were more informative than SSI for identifying superior genotypes under deficit irrigation. SSI tends to favor genotypes with small relative yield changes, which may include low-yielding materials that appear stable simply because they perform poorly in both environments (Fernandez, 1992). In contrast, GMP and HM better captured the performance of ACC_49 and ACC_50 because these indices integrate productivity and stability, identifying genotypes capable of maintaining competitive yields under both favorable and restricted water conditions (Saddiq et al., 2021; Pathan et al., 2023).

This interpretation was reinforced by the water use efficiency results. ACC_49 and ACC_50 combined relatively high WUE with superior grain yield, indicating more efficient partitioning of assimilates toward reproductive sinks (Geerts et al., 2008; Telahigue et al., 2017). ACC_23, despite exhibiting high biomass efficiency, did not achieve proportional grain yield, demonstrating that high vegetative productivity alone does not guarantee agronomic efficiency under deficit irrigation. The key limitation appears to be the inability to maintain reproductive partitioning under sustained water restriction (Dumschott et al., 2022; Mirsafi et al., 2024).

This finding underscores a critical principle for breeding under water-limited environments: selecting genotypes solely based on stability indices such as SSI may favor low-performing cultivars with limited productive potential. In contrast, ACC_49 and ACC_50 balanced productivity, stability, and water-use efficiency, making them more promising candidates for arid production systems (Lupa-Condo et al., 2024).

### 4.4 Spectral phenotyping and multivariate relationships

Spectral phenotyping showed that visible-spectrum indices were more sensitive than NDVI for detecting early responses to deficit irrigation. Indices such as HIV, NGRD, RG, and PSRI responded strongly to changes in pigment composition and canopy color before major structural changes became evident (Gitelson et al., 2002; Ballester et al., 2019). This is expected because visible-spectrum indices capture subtle shifts in chlorophyll and carotenoid balance, whereas NDVI is more closely related to total biomass and tends to saturate in dense canopies (Çolak et al., 2021; Wang et al., 2023). Therefore, visible-spectrum indices appear more suitable for the early detection of physiological stress in quinoa because they detect subtle pigment and canopy color changes before major structural alterations become detectable through NIR-based indices.

The spectral signatures corroborated these differences. Genotypes such as SALCEDO exhibited higher near-infrared reflectance, suggesting better preservation of internal leaf structure and hydration (El-Hendawy et al., 2019; Zhang and Zhou, 2019), whereas ACC_49 displayed changes in the red-edge region that are consistent with fine adjustments in chlorophyll and nitrogen status (Xie et al., 2018; Easterday et al., 2019) These responses suggest that deficit irrigation altered both physiological function and canopy structure, although not uniformly across genotypes.

The PCA further supported this interpretation by separating genotypes according to biomass-related traits, pigment-related indices, and temporal responses. Early and mid-season evaluations showed greater genotype dispersion, indicating stronger genotype-specific responses during the onset of water limitation. Later evaluations converged as plants approached maturity, suggesting that canopy structure became progressively more uniform across treatments. Correlation analysis reinforced the central agronomic message of the study: grain yield was strongly associated with harvest index, grain size, and reproductive traits, whereas WUE was more closely related to biomass accumulation than to yield itself. This indicates that efficient water use during vegetative growth does not necessarily guarantee superior grain production. Instead, yield stability under deficit irrigation depends on maintaining reproductive sink strength and efficient assimilate partitioning. Together, these findings support the integration of spectral phenotyping with conventional phenotyping as a powerful methodological improvement for early and accurate screening of drought-tolerant quinoa genotypes (Araus and Cairns, 2014; Sankaran et al., 2019).

## 5 Conclusions

The present study demonstrated that deficit irrigation significantly affected quinoa performance under the arid conditions of Majes, Peru, while also revealing substantial genetic variability in drought response. This variability enabled the identification of contrasting adaptive strategies and genotypes with greater drought tolerance. Genotypes such as ACC_49 and ACC_50 combined high productivity with favorable tolerance responses, whereas B_JULY and ACC_06 showed greater yield stability associated with conservative physiological behavior under water deficit. Agromorphological and physiological traits related to reproductive maintenance, tissue hydration, and biomass accumulation were key determinants of drought adaptation. Among the evaluated tolerance indices, GMP and HM were more effective than SSI for identifying superior genotypes under contrasting irrigation conditions, while visible-spectrum indices such as HIV, NGRD, RG, and PSRI were more sensitive than NDVI for early stress detection. Spectral and multivariate analyses further confirmed strong associations among yield, grain traits, biomass accumulation, and canopy related vegetation indices. Overall, these findings support the use of integrated agromorphological, physiological, and multispectral approaches for quinoa phenotyping, drought-tolerance screening, and irrigation management in arid environments.

## Data Availability

The datasets presented in this article are not readily available because The dataset is intended exclusively for academic and research purposes. Commercial use is not permitted without prior written authorization. Proper citation of the original publication is required in any derivative work. Redistribution of the dataset to third parties is not allowed without explicit permission. Requests to access the datasets should be directed to MM-A, mmaytaan@unsa.edu.pe.
